# Towards a Harmonized Terminology: A Glossary for Biocide Susceptibility Testing

**DOI:** 10.3390/pathogens11121455

**Published:** 2022-12-01

**Authors:** Szilvia Neuhaus, Andrea T. Feßler, Ralf Dieckmann, Lara Thieme, Mathias W. Pletz, Stefan Schwarz, Sascha Al Dahouk

**Affiliations:** 1 German Federal Institute for Risk Assessment, 10589 Berlin, Germany; 2 Centre for Infection Medicine, Department of Veterinary Medicine, Institute of Microbiology and Epizootics, Freie Universität Berlin, 14163 Berlin, Germany; 3 Veterinary Centre for Resistance Research (TZR), Freie Universität Berlin, 14163 Berlin, Germany; 4 Institute of Infectious Diseases and Infection Control, Jena University Hospital, Friedrich-Schiller-University Jena, 07747 Jena, Germany; 5 Leibniz Center for Photonics in Infection Research, Jena University Hospital, Friedrich Schiller University Jena, 07747 Jena, Germany; 6 Department of Internal Medicine, RWTH Aachen University Hospital, 52074 Aachen, Germany

**Keywords:** biocide susceptibility testing, biocide resistance, antimicrobial resistance, biocide tolerance

## Abstract

Disinfection is a key strategy to reduce the burden of infections. The contact of bacteria to biocides—the active substances of disinfectants—has been linked to bacterial adaptation and the development of antimicrobial resistance. Currently, there is no scientific consensus on whether the excessive use of biocides contributes to the emergence and spread of multidrug resistant bacteria. The comprehensive analysis of available data remains a challenge because neither uniform test procedures nor standardized interpretive criteria nor harmonized terms are available to describe altered bacterial susceptibility to biocides. In our review, we investigated the variety of criteria and the diversity of terms applied to interpret findings in original studies performing biocide susceptibility testing (BST) of field isolates. An additional analysis of reviews summarizing the knowledge of individual studies on altered biocide susceptibility provided insights into currently available broader concepts for data interpretation. Both approaches pointed out the urgent need for standardization. We, therefore, propose that the well-established and approved concepts for interpretation of antimicrobial susceptibility testing data should serve as a role model to evaluate biocide resistance mechanisms on a single cell level. Furthermore, we emphasize the adaptations necessary to acknowledge the specific needs for the evaluation of BST data. Our approach might help to increase scientific awareness and acceptance.

## 1. Introduction

Bacterial antimicrobial resistance (AMR) is a major global threat to food safety and animal and public health. It is caused by the bacterial mechanisms rendering the drugs used to treat infections less effective. About 4.95 million deaths were estimated to be associated with bacterial AMR in 2019, including 1.27 million deaths clearly attributable to AMR [1]. Infection prevention and control through effective hygiene measures is one key strategy to reduce the emergence and spread of multidrug-resistant bacteria [2]. For this purpose, biocides have been used as disinfectants and antiseptics in human and veterinary medicine for decades. Disinfectants are not only applied in the healthcare sector, but also in different industries, for example along the food chain, to ensure product safety [3]. The putative risks associated with the extensive use of biocides, such as bacterial adaptation or the development and spread of AMR, have raised awareness in the scientific community [3]. Nosocomial outbreaks caused by pathogens resistant to the applied disinfectants have been described [4]. To reveal the bacterial adaptation to biocides in laboratory or epidemiological studies, susceptibility testing of bacteria to substances of interest is a prerequisite. The comparison of the study results, however, remains a challenge since neither standardized test procedures nor uniform evaluation criteria are available. In addition, there is no scientific consensus on the use of terminology to report changes in biocide susceptibility. Currently, most original studies investigate the minimal inhibitory concentration (MIC) of biocides as a marker for the susceptibility of bacteria. This approach neglects the fact that biocides are usually used at concentrations far exceeding the MIC. Considering that the minimal bactericidal concentration (MBC) provides information on the lethal effect of a biocide (reviewed in [5]), this value might be more appropriate to identify alterations in bacterial susceptibility. However, not every biocide is applied at lethal concentrations. Instead, concentrations are chosen to inhibit microbial growth. In these cases, MIC determination is suitable. Furthermore, biocides are widely used in formulations containing various ingredients, which may influence product efficacy. Therefore, the susceptibility data of pure substances will not necessarily allow for drawing conclusions on actual product efficacy. Despite these numerous limitations, biocide susceptibility data are indispensable to monitor changes in the susceptibility of bacteria to biocidal substances, including data that are needed to identify bacterial adaptation at an early stage. In addition to the implementation and uniform use of standardized test procedures, interpretive criteria and the terms applied to describe altered bacterial susceptibility to biocides need to be standardized in order to allow for the interpretation and comparison of available study results. 

In our review, we investigated the diversity of terms used to describe the observed changes in bacterial susceptibility to biocidal substances and the variety of criteria applied to interpret findings. For this purpose, we reviewed the current literature in a four-stage process. First, we analyzed original studies performing biocide susceptibility testing (BST) of field isolates from different environments to assess methods of data interpretation. Second, we screened reviews summarizing the knowledge of individual studies on altered biocide susceptibility for a better understanding of currently available broader concepts for data interpretation. Third, we propose interpretive criteria and terms which should be used to categorize biocide susceptibility testing data. Finally, we point out the relevance of biofilm formation for the evaluation of bacterial susceptibility to biocides applied on surfaces.

## 2. Interpretive Criteria and Terms Used to Assess Biocide Susceptibility Data

To conduct an overview of the diversity of interpretive criteria and terms currently applied to assess bacterial susceptibility to biocides, we investigated original studies providing biocide susceptibility data of field isolates from various environments in planktonic form. For reasons of consistency, we restricted our comparative analysis to studies reporting on bacterial MIC values of pure substances. Currently, MIC testing represents the lowest common denominator to evaluate bacterial susceptibility to biocides. To identify as many suitable studies as possible, we conducted a PubMed query with the combined search terms “biocide toleran*”[tiab] OR “biocide resist*”[tiab] OR “biocide suscept*”[tiab] OR “biocide adapt*”[tiab] OR “disinfectant toleran*”[tiab] OR “disinfectant resist*”[tiab] OR “disinfectant suscept*”[tiab] OR “disinfectant adapt*”[tiab] OR “microbicide toleran*”[tiab] OR “microbicide resist*”[tiab] OR “microbicide suscept*”[tiab] OR “microbicide adapt*”[tiab] AND bact* on 1 April 2022. In total, our literature search resulted in 412 publications, including 48 reviews. Screening of titles and abstracts of the 364 original studies revealed 156 publications within the scope of our review. Subsequently, the methods section of each publication was evaluated to identify reports on MIC data to pure biocidal substances (Table S1). Finally, 84 studies were analyzed for the interpretive criteria and terms used to describe bacteria with increased MICs.

Table 1 summarizes the variety of classification schemes available for biocide susceptibility data and gives an overview of the terminology in the field. In addition, Table S1 provides the terms and interpretive criteria published in the original studies. The interpretive criteria applied for the classification of MIC data varied considerably. Overall, a comparative analysis of own datasets (*n* = 25), a consideration of previously published thresholds (*n* = 16), and a comparison with reference strains (*n* = 7) or thresholds derived from biocide concentrations in products (in-use concentrations, *n* = 8) served as benchmarks.

Each of the abovementioned interpretive criteria was used in several original studies. However, the study results were described by using different terms. Twenty-five studies, for example, compared MIC data of different groups of bacteria within their own datasets to identify isolates with increased values. Depending on the study, such isolates were called resistant, tolerant, reduced susceptible, non-susceptible, or were designated as non-wildtype isolates. In sixteen studies, MIC changes were described without final assessment. The included studies provided an insight into the variability of terms and their inconsistent use, which hampers the comparison and interpretation of results.

To conduct an overview on the various concepts to classify bacterial susceptibility to biocides, we investigated reviews and opinions regarding their use of terms and the applied interpretive criteria. These summary publications usually interpret the data of original studies in a broader context, outlining their relevance with regard to bacterial adaptation to biocides as well as the development and spread of antimicrobial resistant bacteria due to the application of biocides as disinfectants or antiseptics. For this purpose, authors initially need to introduce a common basis for the interpretation of study results. We considered all reviews and opinions of our PubMed query and included those publications that summarized and/or discussed findings on phenotypic biocide susceptibility data of bacteria in planktonic form. In addition, the reference list of each manuscript was screened for further relevant documents. In total, we included 48 reviews and opinions. Indeed, several authors pointed out the inconsistent use of the terms *biocide resistance* and/or *biocide tolerance* and discussed the problems that may arise due to the lack of interpretive criteria, such as labeling bacterial isolates with only minor changes in their susceptibility to biocides as *biocide resistant* [5,90,91,92,93,94]. In this context, most reviews provided definitions for the terms used. Compared to the original studies, the interpretive criteria are less diverse. There are mainly three approaches to categorize bacteria according to their *biocide resistance*:

1. Comparison to in-use concentrations: *biocide resistance* is strictly defined as the failure of bacterial killing or growth inhibition by the use of biocide concentrations attained in practice [3,93,95,96,97,98,99,100,101,102,103,104];
2. Comparative data analysis on the population level: *biocide resistance* refers to isolates that are neither killed nor inhibited by a concentration at which the majority of isolates of the respective species are killed or inhibited [3,97,102,105,106,107,108];
3. Comparative data analysis on the bacterial cell level: *biocide resistance* refers to bacterial cells of an individual isolate that are neither killed nor inhibited by a concentration effective against the majority of cells of this isolate [3,5,102].

Interestingly, some reviews provide up to three different definitions for the term *biocide resistance*, a fact pointing towards the lack of scientific consensus on this topic [3,5,97,102]. The term *reduced susceptibility* is frequently used to describe increased MIC and/or MBC below in-use concentrations [5,96,98,100,109,110,111,112]. The term *biocide tolerance* commonly describes decreased susceptibility to a biocide, which has evolved by adaptation [95,99,105,106,113,114,115]. In some publications, *biocide tolerance* describes the development of increased MIC values below in-use concentrations (synonymous to *reduced susceptibility*) [3,94,116]. In several reviews, authors distinguish *low-level resistance* (biocide MIC below in-use concentrations) from (*high*-*level*) *resistance* (biocide MIC above in-use concentrations) [117,118,119,120]. Other publications introduce the terms *nonsusceptibility* or *insusceptibility* to describe increased MIC values [99,120,121,122,123,124]. Some authors restrict *insusceptibility* to the intrinsic properties of the organism [3,93,105]. Interestingly, the reviews sometimes did not introduce a definition of the applied terms [110,111,113,125,126,127,128,129,130,131,132,133,134,135]. Overall, our literature analysis clearly shows the variability of concepts for the interpretation and categorization of biocide susceptibility data from multiple original studies. Even though the applied interpretive criteria and introduced terms are less diverse in reviews compared to original studies, the inconsistency observed reflects the uncertainty of the scientific community and emphasizes the need for unambiguous definitions.

## 3. Antimicrobial Susceptibility Testing: A Blueprint for Biocide Susceptibility Terminology?

The use of standardized test procedures and the definition of interpretive criteria are important prerequisites to identify and classify changes in bacterial susceptibility to antimicrobial agents. While these requirements are missing for BST, for antimicrobial susceptibility testing (AST) they are met. The AST procedures mainly follow the well-established standards of the Clinical and Laboratory Standards Institute (CLSI) and the European Committee on Antimicrobial Susceptibility Testing (EUCAST). These standards define the essential requirements for the methods, materials, and practices that have to be applied in a non-modified form [136]. AST may be performed by using various methods, such as agar disk diffusion, E-test, broth microdilution, and broth macrodilution or agar dilution in addition to automated systems. The obtained results allow the classification of the bacteria tested into different categories, which, however, depend on the applied interpretive criteria. There are two main types of interpretive criteria: clinical breakpoints (CBPs) and epidemiological cut-off values (ECOFFs or ECVs).

A CBP is specific for a combination of an antimicrobial agent, bacterial species, site of infection, and human or animal species and depends on the dosage of the antimicrobial agent applied. The AST results based on CBPs provide guidance to medical doctors and veterinary practitioners in their choice for the most efficacious antimicrobial agent, dosage, route of administration (orally or per injection), and administration scheme, i.e., intermittent versus prolonged or continuous infusion. The categories for clinical breakpoints according to CLSI are: susceptible (S), susceptible-dose dependent (SDD), intermediate (I), resistant (R), and nonsusceptible (NS) [137]. Susceptible isolates are inhibited by the concentrations of an antimicrobial agent usually achievable at the site of infection when the dosage recommended for treatment is administered. In the category SDD, susceptibility of an isolate depends on the dosing regimen that is used in the patient. To achieve clinical efficacy, a dosage that results in a higher drug exposure than that recommended to treat susceptible isolates is necessary. The category SDD is currently limited to applications in human medicine. Isolates in the category intermediate (I) may have lower response rates than the susceptible isolates. Resistant isolates are not inhibited by the concentrations of the agent usually achievable with regular dosage schedules and/or AST data fall in a range in which specific microbial resistance mechanisms are likely, and clinical efficacy of the antimicrobial agent against the isolates has not been reliably shown in treatment studies. The category nonsusceptible (NS) is used for isolates for which only a susceptible breakpoint is defined because of the absence or rare occurrence of resistant isolates. Isolates for which AST data are outside the range indicated for the susceptible breakpoint should be reported as nonsusceptible. In contrast to CLSI, EUCAST proposes only three categories for clinical breakpoints: S—susceptible, standard dosing regimen; I—susceptible, increased exposure; and R—resistant (https://www.eucast.org/newsiandr/, last accessed: 31 October 2022).

The AST results based on ECOFF values provide an insight into the changes of MIC distributions and may help to detect variations in the bacterial population, such as newly emerging resistance properties. When applying ECOFF values, the categories are wildtype and non-wildtype. The wildtype subpopulation includes the majority of the bacteria in a tested population. Considering that these bacteria do not possess acquired resistance mechanisms, they show lower MICs or larger zone diameters. In contrast, the bacteria of the non-wildtype subpopulation harbor acquired resistance mechanisms and, as a consequence, show higher MIC values and smaller zone diameters. An ECOFF value is specific for the combination of an antimicrobial agent and a bacterium. ECOFF values are defined by means of aggregated datasets fulfilling criteria outlined in the EUCAST standard operating procedure (SOP) 10.1 [138].

It is important to consider the application fields and limitations of both interpretive criteria. CBPs are established in consideration of in vivo (pharmacokinetics, pharmacodynamics, and clinical outcome data) and in vitro data (MIC distributions/zone diameter distributions) to provide guidance for systemic antimicrobial medication [139]. They are not suitable for topical applications. ECOFFs are exclusively based on phenotypic data (MICs or zone diameters) to identify non-wildtype subpopulations harboring horizontally acquired or mutational resistance mechanisms. In contrast to CBPs, ECOFFs have not been investigated for their clinical relevance. Both interpretive criteria from AST, CBPs, and ECOFFs, are frequently used for the interpretation of biocide susceptibility data in a modified form. In contrast to AST, data are not limited to MIC values and zone diameters. MBC values, which consider the lethal effect of bactericidal biocides, are additionally evaluated. The evaluation of susceptibility to pure biocidal substances based on comparison to in-use concentrations resembles the interpretive criterion of CBPs in AST.

However, such a categorization does not provide information on the efficacy of disinfectants or antiseptics containing the substance of interest. Additional factors such as application specifications (e.g., exposure time and dosage) and bacterial lifestyle (planktonic vs. sessile) need to be considered. This fact contrasts sharply with the available concept for the interpretation of AST data, which provide guidance for the choice of the antimicrobial agent that is the most efficacious in systemic treatment. It is further essential to consider the consequences arising from the choice of in-use concentrations as an interpretive criterion for biocide resistance. As active ingredients of disinfectants and antiseptics, biocides are predominantly applied in concentrations exceeding the bacterial MIC and MBC by far. Thus, the identification of isolates with MICs and MBCs above in-use concentrations should be currently regarded as a rare phenomenon, as recently reported for the combination of *Pseudomonas aeruginosa* and benzalkonium chloride [30].

Some publications introduce (tentative) ECOFF values for various combinations of biocides and bacterial species [28,66,84]. Compared to AST, these cut-off values have to be considered as preliminary because they do not fulfill the essential criteria that are outlined in the EUCAST SOP 10.1 [138], such as use of a standardized method and aggregated datasets to cope with inter-laboratory variability. Considering that the AST SOP is already available, it could help to define the necessary specifications for the identification of ECOFF values for various combinations of bacterial species and biocides.

## 4. Introduction of a Glossary

Antimicrobial agents and biocides reveal bacteriostatic and/or bactericidal activities. Consequently, similar terminology defined by similar interpretive criteria are desirable wherever possible, to avoid confusion in the scientific community and among end users, such as physicians, hygienists, veterinarians, and farmers.

In addition, bacterial susceptibility to biocides is frequently evaluated along with susceptibility to antimicrobial agents, for example to investigate the relevance of co- and cross-resistance for both substance groups. In this context, the use of different interpretive criteria and terminology hampers the discussion. Nonetheless, the differences in the application of biocides prohibit the direct transfer of concepts available for identification and interpretation of bacterial susceptibility to antimicrobial agents. In addition to MIC determination—the central method to evaluate susceptibility to antimicrobial agents—the MBC provides additional valuable information for biocidal substances used as the active ingredients of disinfectants and antiseptics, because this value considers the lethal effect of a biocide. Thus, the terminology and interpretive criteria applied to evaluate bacterial susceptibility to biocidal substances need to be suitable for MIC and MBC data. Table 2 provides an overview of the available interpretive criteria and terms used for the evaluation of AST data and the proposed interpretive criteria and terms for BST.

In line with the application-dependent interpretive criterion CBP for AST, we propose that in-use concentrations should serve as the basis for the identification of bacterial resistance to biocidal substances. Accordingly, biocide resistance refers to isolates that are neither killed nor inhibited by concentrations attained in practice. The interpretive criterion should include the categories resistant and susceptible. Resistant isolates exhibit MIC and/or MBC values exceeding the concentrations attained in practice, while MICs and/or MBCs of susceptible isolates are below the concentrations attained in practice. According to this definition, biocide resistance should be considered a rare phenomenon to date. As already outlined in the previous section, the identification of bacterial resistance to a specific substance does not allow conclusions to be drawn on disinfectant efficacy [30,31]. Nonetheless, the BST results may influence the product choice, for example in the case that the observed susceptibility is close to the concentration attained in practice.

With most isolates being susceptible to biocidal substances when the application-dependent criterion is used, an additional concept is needed for the evaluation of altered biocide susceptibility below in-use concentrations. Epidemiological cut-off values have the power to separate bacterial species into isolates with and without acquired resistance based on their phenotype [140], and they are highly suitable for surveillance of spatial and temporal shifts of MIC and/or MBC values to substances of interest. Hence, we propose epidemiological cut-off values as a further interpretive criterion for the evaluation of biocide susceptibility data. In accordance with the concept for AST, this interpretive criterion allows a distinction between wildtype and non-wildtype. However, for BST, epidemiological cut-off values of both MICs and MBCs should be defined. Wildtype and non-wildtype subpopulations usually show bimodal distributions of biocide MICs or MBCs. The isolates in the non-wildtype category exhibit higher MICs or MBCs than the ones in the wildtype category. We consider the use of epidemiological cut-off values most suitable for the surveillance of development and spread of isolates with elevated MICs/MBCs to biocidal substances.

A prerequisite prior to the implementation of both proposed interpretive criteria will be the successful establishment of standardized test procedures. Frequently, original studies perform MIC testing for biocides following CLSI and EUCAST standards with minor modifications. However, even minor modifications may influence test results, impeding the comparative analysis of datasets [141]. The use of a standard in a non-modified form will help to create comparable MIC datasets, fostering the definition of ECOFFs for specific combinations of bacterial species and substances. The EUCAST SOP 10.1 [138] and the EUCAST ECOFF finder are also useful tools to establish MIC ECOFFs as interpretive criteria for BST. Even with validated laboratory procedures for MIC testing in place (such as [142,143,144]), quality controls are urgently needed in order to verify test results. Only recently, QC ranges for various combinations of ATCC reference strains and selected substances have been determined in an interlaboratory trial [144]. As previously mentioned, MBC values provide additional important information to evaluate bacterial susceptibility to bactericidal biocides. Therefore, standard procedures for MBC testing need to be established as well. Different MBC detection methods are available [145,146]. However, in contrast to MIC testing the direct transfer from methods with widespread use, which are based on years of experience, is not possible.

## 5. Biofilm Formation: A Crucial Component for the Evaluation of Bacterial Susceptibility to Biocides

The BST of planktonic bacteria is mandatory to gain insights into acquired resistance mechanisms on a single cell level. However, evaluating the efficacy of biocides, especially surface disinfectants, should additionally be based on their activity against bacterial biofilms. Biofilms are microbial communities consisting of aggregated bacteria surrounded by a polymeric matrix, which can form upon contact with abiotic or biotic surfaces [147]. Bacterial biofilm contaminations are commonly found on medical instruments and on surfaces in clinical or industrial settings. The efficacy of biocides active against planktonic bacteria is diminished by e.g., biofilm diffusion barriers depending on the degree of matrix maturation and biofilm volume-to-surface ratio, or phenotypic adaptations of inner biofilm cells as a result of arising sublethal concentrations of disinfectants [148,149]. This effect is described as biofilm-mediated tolerance or phenotypic resistance, in contrast to inherited resistance mechanisms on a single cell level due to genetic alterations [150]. An inference of BST results from planktonic to biofilm-embedded cells is, therefore, not possible.

However, neither standardized test procedures nor uniform evaluation criteria for AST and BST of biofilms are currently available, impeding the comparison of study results. As an analogy to MIC and MBC, the two endpoint parameters minimal biofilm inhibitory concentration (MBIC) and minimal biofilm eradication concentration (MBEC) have been proposed by researchers to guide the treatment of biofilm-associated infections [151]. While MBIC describes the lowest concentration of an antimicrobial substance inhibiting the time-dependent increase in the mean number of viable cells in a biofilm, the MBEC refers to the lowest concentration capable to partly reduce (3 log_10_ reduction in CFU/mL) or completely eradicate the viable cells of the biofilm. However, these definitions are differentially perceived, used, and interpreted by the scientific community, mainly due to the lack of linked SOPs defining: i) the method and duration for biofilm growth, ii) the antimicrobial exposure time, iii) the analysis method, and iv) the untreated reference biofilm to determine the effect size, i.e., the biofilm inhibiting (MBIC) or reducing (MBEC) effects [152]. Implementing the standardized testing procedures for biofilm susceptibility testing is extremely challenging due to the strong dependency of biofilm composition and structure on the environment, resulting in a high heterogeneity of primarily similar bacterial biofilms. The profound differences between in vitro and in vivo biofilms have led to a poor clinical validity of current biofilm susceptibility tests, which is why neither the EUCAST nor CLSI has established SOPs for MBEC and MBIC determination and the respective interpretive criteria [153].

This heterogeneity is also reflected in the BST of biofilms. The very same biocide compound showed different levels of efficacy in isolate-identical biofilms depending on the hydration status of the biofilm and the chosen biofilm model, i.e., a dynamic flow reactor system or a static microtiter plate model [154]. Both model systems have been suggested for the standardization of BST [155,156,157,158], raising the question of which model is representative for the “real-world” biocide efficacy. In addition to an appropriate biofilm model, a representative evaluation method, e.g., a colony forming unit determination, the staining of biofilm biomass or the measurement of metabolic activity, needs to be standardized [159,160,161].

Provided standardized and reproducible SOPs for the determination of MBEC and MBIC values of individual biocide–bacterial biofilm combinations have been established, these values, as for planktonic bacteria, could be compared to the in-use concentrations of biocides to categorize the isolates’ biofilm as tolerant or susceptible to the respective biocide. It remains arguable whether the term “tolerant” (to strictly stick to the biofilm terminology) or “resistant” (in analogy to BST of planktonic bacteria) should be used. Nevertheless, strongly enhanced MBIC/MBEC values, compared to the MIC/MBC values, are expected, possibly near or even above biocide in-use concentrations. To measure the discrepancy in biocide susceptibility between planktonic and biofilm cells, the coefficients “Rc” and “Rt” have been applied, presenting the ratio of the concentrations or time required to achieve the same reduction in the planktonic and the biofilm population [149].

## 6. Conclusions

The unambiguous classification of bacterial susceptibility to biocides is a prerequisite for clear and comparable presentation of study results and the interpretation of available data. For this purpose, a harmonized terminology and methodological standards are indispensable. To evaluate resistance mechanisms on a single cell level, the well-established and approved concepts of AST should be the role model in order to increase scientific acceptance. Of course, adaptations are necessary to acknowledge specific needs for the evaluation of BST data. MBCs provide additional important information for assessing bacterial susceptibility to biocides and should be reported along with MICs. The main future tasks include the implementation of MIC and MBC ECOFFs to interpret the BST data for planktonic cells. Despite the difficulties in the standardization of the BST of biofilms and the lack of an AST blueprint, the BST of biofilms in addition to the BST of planktonic cells is essential to adequately evaluate disinfectant efficacy.

## Figures and Tables

**Table 1:** A summary of the interpretive criteria and terms applied for the classification of biocide susceptibility data in original studies.

		Interpretive Criteria for Classification (Number of Publications)				
Terms	Number of Publications	In-Use Concentrations	Own Dataset	Published Thresholds	Reference Strain	Unclear
MIC description	16 [6,7,8,9,10,11,12,13,14,15,16,17,18,19,20,21]					
resistance	28 [22,23,24,25,26,27,28,29,30,31,32,33,34,35,36,37,38,39,40,41,42,43,44,45,46,47,48,49]	7	7	7	1	6
tolerance	23 [50,51,52,53,54,55,56,57,58,59,60,61,62,63,64,65,66,67,68,69,70,71,72]	1	10	6	2	4
reduced susceptibility	8 [73,74,75,76,77,78,79,80]		4	1	3	
wildtype/non-wildtype	4 [81,82,83,84]		3	1		
nonsusceptibility	1 [85]		1			
tolerance and resistance	4 [86,87,88,89]			1	1	2

**Table 2:** The interpretive criteria and terms used for the evaluation of AST data and the proposed interpretive criteria and terms for biocide susceptibility testing of planktonic cells.

Antimicrobial Susceptibility Testing		Biocide Susceptibility Testing	
Definitions	Interpretive Criteria	Proposed Definitions	Interpretive Criteria
**Clinical resistance** Isolates are not inhibited by the concentrations of the agent usually achievable with normal dosage schedules at the site of infection and/or test results fall into the range in which specific microbial resistance mechanisms are likely, and clinical efficacy of the agent has not been reliably shown.	Clinical breakpoints	**Resistance (application-related)** Isolates are neither killed nor inhibited by a biocide concentration attained in practice.	In-use concentrations
**Wildtype/Non-wildtype** Bacterial populations are separated into those without and with acquired resistance mechanisms based on their phenotypes.	Epidemiological cutoff values	**Wildtype/Non-wildtype** A non-wildtype isolate is neither killed nor inhibited by a biocide concentration at which the majority of isolates of the same species are killed or inhibited.	Epidemiological cutoff values

## Data Availability

The data presented in this study are available in the article and the Supplementary Materials.

## Supplementary Materials

The following supporting information can be downloaded at: https://www.mdpi.com/article/10.3390/pathogens11121455/s1, Table S1: The applied terms and interpretive criteria extracted from original studies.
